# A protocol for a pilot cluster randomized control trial of e-vouchers and mobile phone application to enhance access to maternal health services in Cameroon

**DOI:** 10.1186/s40814-020-00589-y

**Published:** 2020-04-14

**Authors:** Miriam N. Nkangu, Patrick M. Okwen, Lawrence Mbuagbaw, Donald K. Weledji, Janet Hatcher Roberts, Sanni Yaya

**Affiliations:** 1 School of Epidemiology and Public Health, University of Ottawa, Ottawa, Canada; 2 Effective Basic Services (eBASE) Africa, Bamenda, Cameroon; 3 Department of Health Research Methods, Evidence and Impact, McMaster University, Hamilton, Canada; 4 Biostatistics Unit, The Research Institute, St Joseph’s Healthcare Hamilton, Hamilton, Canada; 5 Centre for the Development of Best Practices in Health, Yaounde, Cameroon; 6 SPRL Donwels System, Brussels, Belgium; 7 Health Promotion Alliance Cameroon (HPAC), Yaounde, Cameroon; 8 School of International Development and Global Studies, University of Ottawa, Ottawa, Canada; 9 WHO Collaborating Center for Knowledge Translation and Health Technology Assessment in Health Equity, Ottawa University, Ottawa, Canada; 10 Bruyere Research Institute, Ottawa, Canada

**Keywords:** mHealth, Vouchers, Maternal mortality, Family planning, Reproductive health, Cluster randomized control trials, Qualitative research, Geographic information system, Pilot, Feasibility

## Abstract

**Background:**

Cameroon still has relatively high maternal mortality rate (MMR) of 596/100,000 live births. Approximately 40% of births are unattended by skilled healthcare personnel with high out-of-pocket expenditures. Poor resource allocation, poorly functioning referral systems, long trekking distances to health facilities, all of which lead to low rates of use of maternal health services.

**Objectives:**

The aim of this pilot study is to explore perception and acceptability of mobile health (mhealth) and e-voucher and to determine the feasibility of conducting a large cluster randomized trial to determine the effects of combining e-vouchers and a mobile application compared with usual care in improving access to and use of maternal health services.

**Methods:**

This is a multimethod study that comprises two phases. The first phase is the development of the mobile phone app, which includes a qualitative formative study through in-depth key informant interviews and focus group discussions. The second phase is a cluster randomized control trial assessing the combination of e-vouchers and a mobile application compared with usual care in improving access to and use of maternal health services. Feasibility will be determined based on evaluating randomization, contamination, enrollment rate, complete follow up, compliance rate, success in matching data from different sources, and data completeness.

**Ethics and discussion:**

Ethics approval has been granted, and the trial has been registered in the Pan-African Clinical Trials Registry. We will disseminate our findings through peer-reviewed manuscripts and conference presentations. Findings from this study will inform the design and conduct of a larger randomized trial.

**Trial registration:**

PACTR201808703097367. The trial on the Pan African Clinical Trials Registry.

## Background

Despite tremendous efforts to improve maternal and child health in developing countries, there are indications that low-income individuals have not benefited much in the areas of maternal and child health [1]. In Africa, most women are unable to seek health care due to high out-of-pocket costs [1–12]. Systematic reviews on voucher interventions in addressing maternal mortality have shown that vouchers can be effective in improving access to and utilization of maternal health services, especially in poor and underserved communities [11–26]. Vouchers typically grant purchasing power to low-income individuals who may otherwise be ignored in the market due to their lack of funds or knowledge of goods and services [18, 19].

Cameroon is a lower-middle income country with a relatively high maternal mortality rate (MMR) of 596/100,000 live births [2–5]. Approximately 40% of births are unattended by skilled healthcare personnel [2–9], with approximately 35% of deliveries occurring at home [8]. Approximately 85% of women in Cameroon, especially rural women, have never received family planning education from a healthcare professional [8]. Women of reproductive age continue to die from pregnancy-related causes in Cameroon [2–5]. One potential factor associated with this stagnant progress toward achieving a considerable decline in maternal mortality is the low percentage (64%) of women delivering in hospitals [2–9]. This finding has been attributed partly to the policies that affect the healthcare delivery system, especially with respect to resource allocation and poorly functioning referral systems, coupled with the introduction of user fees in the public health sector [2–9], resulting in an out-of-pocket per capita expenditure of 94.6% [2].

In 2011, a performance-based financing (PBF) pre-pilot program was initiated in Cameroon, covering four health districts, with a focus on maternal and child health and communicable diseases, especially in certain vulnerable groups such as the poor, women, children, and people living with human immunodeficiency virus (HIV)/acquired immunodeficiency syndrome (AIDS) [2]. In July 2012, the program was expanded to the northwestern and southwestern regions of Cameroon. PBF is a strategy in which providers are paid based on their output with respect to a predefined quality standard to improve the quality of care delivery, especially for maternal health services [2]. Cameroon has adopted PBF as a national strategy toward achieving universal health coverage.

Digital health is currently revolutionizing the delivery of health services, most importantly reproductive, maternal, newborn, and child health (RMNCH) services, in developing countries [27, 28]. Systematic reviews have shown mobile phones to be effective in delivering maternal health education, advice, and support [27, 28]. However, reported challenges have been noted in the utilization among nonliterate groups, especially in rural areas. In addition, there are challenges in accessing RMNCH services during emergencies, especially in hard-to-reach communities with difficult terrains, little or no constant power supply, and poor networks. In some areas, there are practically no motorable roads; thus, women trek considerable distances before reaching a health center or reaching any means of transportation.

Our aim is to incorporate vouchers (a demand-side strategy) and mobile phone applications into the healthcare delivery system to complement the newly implemented PBF in Cameroon, which has the potential to boost the quality of provider performance as a supply-side mechanism to enhance sustainability. This study seeks to address whether implementation of vouchers, concurrently with PBF, will help reduce the barriers associated with delays in accessing maternal health care services. This will also inform subsequent research aimed at scaling-up e-vouchers and mobile phone interventions to improve maternal health.

### Objectives

The aim of this study is to explore perception and acceptability of using mhealth and e-voucher in delivery RMNCH and to determine the feasibility of conducting a cluster randomized trial to increase access and utilization of antenatal care, skilled birth delivery, and family planning awareness among rural poor women in Cameroon using targeted e-vouchers and mobile phone applications. This study integrates e-vouchers within the PBF platform concurrently to deliver maternal care services. The study will assess the feasibility of the processes involved, management of resources, mobile phone application software, and resources used.

## Methodology

### Theoretical framework

Three conceptual frameworks are considered in this study: The first framework is the three delays model by Thaddeus and Maine (1998), which maps out key factors (delays) at various stages that may affect the intervention [29]. This model was considered in the design of the platform for the mobile phone applications and to explore the experiences of the community to map out contextual barriers to accessing reproductive maternal and child health services and to inform or modify aspects of interventions and features of the mobile application where applicable. The second framework is the Anderson behavioral theory on healthcare utilization; this will assess health-seeking behavior and utilization of RMNCH [30], which is a function of three characteristics [30].The third framework is the transtheoretical model [31, 32], which we will use to understand and map out the five stages of health behavior change within the community in relation to family planning. This framework and these stages will be used to develop social marketing messages for the family planning intervention, which will be based on the community’s needs [31–34].

**Fig. 1:**
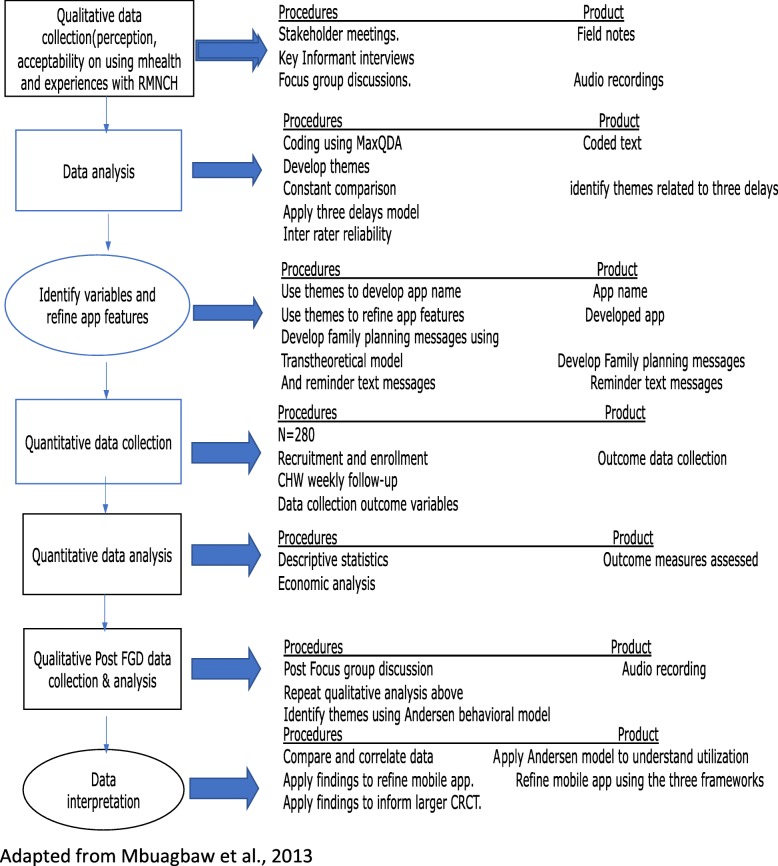
Project framework

The rationale for using the three frameworks above is based on the hypothesis that access to services does not necessarily translate to utilization of services. Thus, the three delays model is used to map out the contextual barriers in accessing RMNCH services at different stages, and to guide the development of the mobile application and help inform specific or relevant areas of intervention and modification of the mobile app. The transtheoretical model is specific to understanding the behavioral change process and stages of the family planning aspect to help develop the messages that will be incorporated within the family planning icon, while the Anderson behavioral model will be used to understand the process of the intervention and utilization of services at different levels (see Fig. 1).

### Study design

The study is a multimethod study that follows an exploratory sequential design [35–37]. The study is comprised of two phases: a formative study through key informant interviews and focus group discussions, followed by a cluster randomized control trial including a follow-up focus group discussion for post-intervention groups. The rationale for using this exploratory design is that the formative phase will inform some aspects of the intervention from the outset and the mobile application. This is also an important criterion, as recommended in the guidelines for mhealth evidence reporting and assessment [38]. In addition, the data from the formative study will provide supportive information and findings that can be easily generalized within the context.

### Setting, context, and population

The intervention will be carried out in the Bali and Ndop Health Districts. These health districts are in the northwest region of Cameroon and are rural areas. The population sizes of these health districts are as follows: Bali: 73,614, Ndop: 218,505.

### Study districts

The study districts will be purposefully selected because the district medical officers approved of the implementation of the intervention trial in their respective districts. We planned to stratify PBF and non PBF districts into intervention and control districts; however, with the adoption of PBF as a national strategy, the project may take a pragmatic approach if the non-PBF district eventually gets enrolled into PBF.

#### Phase I: formative study for intervention development

##### Objective

The objective of this phase is to explore the community’s experiences in accessing RMNCH and their perception and acceptability of using mhealth in the delivery of RMNCH care. This phase will explore the perspectives of pregnant women, relevant stakeholders, and health care providers.

##### Sampling

i. Sample size and enrolment

Purposive sampling will be used to initially recruit participants for the key informant interviews and focus group discussions. The snowballing technique will be used to reach out to pregnant women who do not have access to the health center. Sampling in each group will continue until emergent themes begin to appear [39–41].

The study aims to explore the following outcomes—community perception and acceptability of using mobile phones and e-vouchers in the delivery of maternal health services and their experiences. The outcome will help to refine features of the app and to understand and inform how changes in behavior are initiated and how they can be sustained.

ii. Enrolment

Women who have given birth, pregnant women, and/or breastfeeding mothers will be eligible for the KI interviews, in addition to key informants as identified within the community during stakeholder meetings. A minimum of six and maximum of eight participants (both men and women) will be enrolled in each focus group discussion. Participants for focus group will be recruited using a theoretical sampling approach.

iii. Data generation

The formative study will explore the following sociocultural and structural factors associated with the access to and utilization of maternal health services: the barriers and facilitators during pregnancy; the participants’ perception of antenatal care, skilled birth delivery, and family planning; their experiences with using maternal health services; and their perception of using mobile phones in delivery care. Components as well as the sociocultural and structural factors that facilitate or hinder the providers’ quality of delivery care will also be assessed. Focus group discussions including 6–8 members will be conducted separately for both men and women. The post-intervention focus group will explore their experiences in using the mobile phones and their difficulties, and feedback and recommendations will be solicited to improve and refine the mobile application.

iv. Qualitative data analysis approach

The data analysis will include a directed content analysis, which starts with a theory (for example, the three delays model) or relevant research findings as guidance for the initial codes [39–41]. Transcribed text that describes any of the three delays will be highlighted, and a deductive approach will be used in all highlighted text, which will be compared and sorted using all predetermined categories of all the delays. Specifically, for family planning, we will upload transcripts to MaxQDA (https://www.maxqda.com/) and develop codes using grounded theory approach. Next, we will visualize all the data on MaxQDA using maps, emerging themes and modeling, and we will retrieve and export results as code books, maps, or themes.

We will use results from the formative study to develop a social marketing strategy for behavior change using the transtheoretical model [31–34] to map out the stages of change for the uptake of family planning and antenatal care services. Finally, we will identify the five levels of change using barriers and enablers identified from key informants and focus group discussion narratives and use these findings to design binary pictorial decision aids for the uptake of family planning and antenatal care services.

#### Development of the application and theory of change for the intervention

The development phase of the application involved continuous consultations with relevant stakeholders, including health providers, district medical officers, and community members within the specific context and literature reviews. These processes involved key informant interviews and focus group discussions to inform the intervention and refine the features, as well as to ensure project buy-in and to identify relevant contextual barriers and conditions to facilitate the intervention and ensure availability of the necessary resources to implement the intervention to achieve the desirable outcomes. The theory of change is attached as supplemental material (suppl figure 1) with examples of assumptions, the rationale, and some aspects of the intervention highlighting the various pathways of the intervention (suppl table 1). The final theory of change (ToC) will be updated at the end of the project after consultation with relevant stakeholders and user feedback evaluation including lessons learnt during the implementation processes.

#### Phase II: protocol for cluster randomized trial of electronic vouchers and a mobile application

##### Objective

To determine the feasibility of conducting a cluster randomized trial to increase access and utilization of antenatal care, skilled birth delivery, and family planning awareness by assessing the processes involved, management of resources, the mobile phone application software, and resources used.

i. Stratification and randomization

We will stratify health areas according to their equity scores (a higher equity score defines how remote or rural the area is, the distance from the district health service, and the enclaved nature of the topography with poor accessibility). Therefore, health areas with equity scores of ≥ 40 in the two participating health districts (Bali and Ndop) will be included. The second stage of stratification involves health areas with equity scores of ≥ 40 with a medicalized health center. All health areas with equity scores of ≥ 40 with no medicalized health center will be excluded at this stage. A medicalized health center implies the presence of a physician. Three health areas in Ndop meet the inclusion criteria with a ≥ 40 equity score and a medicalized center, namely Bambalang, Babesi, and Balikumbat. However, considering that the study requires two health areas, one for intervention and one for control, Balikumbat (equity score 42) will be excluded because it has a lower equity score than Bambalang (equity score 47) and Babesi (equity score 47). Bambalang and Babesi will be further randomized into a control and intervention health area via computer number generation. In the Bali district, Bosa and Nakka had the highest equity scores and had a medicalized center.

**Fig. 2:**
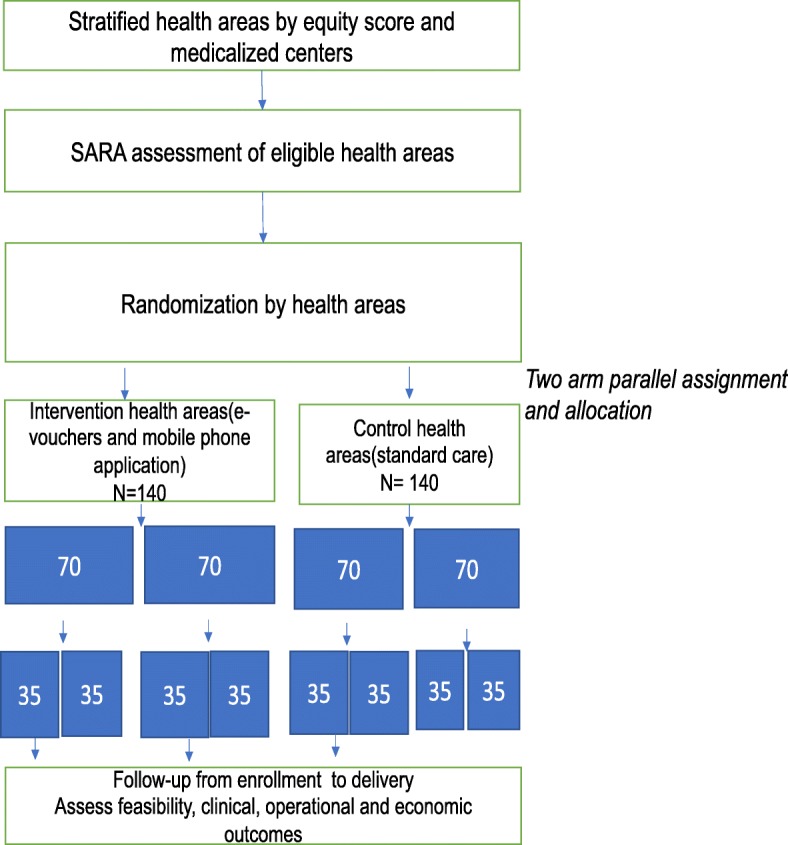
Graphical presentation of CRCT

After stratification, a health facility assessment will be conducted to ensure the quality of health facilities for the intervention using the WHO Service Availability and Readiness Assessment (SARA) tool [42]. Health facilities will be assessed using this tool based on the context of Cameroon to ensure the quality of the services to be delivered and to ensure that all facilities are of equal standard and have the minimum required services to deliver the intervention, followed by the randomization of health areas into intervention and control groups using computer number generation (Fig. 2).

## Participants

The study is open to women aged 15–49 (with the current report of underage pregnancy ages of < 15, we anticipate adjusting the age to 13–49 years because this age group is not only vulnerable but also at high risk). Participants must also be ≤ 4 months pregnant and fall within the poverty scale of the study. Any comorbidity that is present at the time of the study that has a direct and/or indirect effect on antenatal care or the life of the woman or her unborn child will be treated appropriately as emergencies within the study (in the intervention group). This step will be performed after the randomization of the health areas into intervention and control groups. Participants in both groups will regularly interact with community health workers.

## Eligibility criteria

Inclusion criteria

- Women aged 13–49 years, ≤ 4 months pregnant
- Resident in the study area during the entire pregnancy
- Provision of informed consent for study participation
- Meeting the income bracket as defined by the tool that will be employed in the study

Exclusion criteria

- Above the income bracket as defined in the study
- Pregnancy beyond 4 months
- Refusal to provide informed consent

The rationale for excluding women who are more than 4 months pregnant is to allow time to study the outcome of the intervention. The rationale for setting the minimum age of 13 years is due to the current outbreak of teenage pregnancy in these districts (www.ebaseafrica.org) because of the current educational crisis resulting from political instability. The ethics board approved of this age with an accompanying assent form.

## Sample size

The estimated sample size for the anticipated larger study was calculated using clinical outcome variables and using two-sided Z test statistics [43]. The final sample size calculation was computed using WINPEPI by Abramson (2004) [44]. The sample size calculation assumed and estimated that 20% of the individuals in the poorest quintile do not receive more than two antenatal care visits and are less likely to deliver by a skilled birth attendant in Cameroon [2–8]. Previous studies reported an approximately 10–15% increase in the use of antenatal care and a 10% increase in the use of skilled delivery [18–26]. Thus, the study assumes a 15% difference compared with the control assuming an inter-cluster coefficient of 0.001 [26, 45, 46], with four clusters. The study will need a sample size of 160 in each arm and a total of 320 individuals to obtain a 5% significance level and 80% power, assuming a loss to follow up of approximately 5%. The sample size when inflated yields 168 in each arm, a total of 84 in each group and a total of 42 in each cluster. Considering that this is a pilot feasibility study, the final sample size was determined by applying the criteria described by Wittes and Brittain (1990) [47] and Teere et al. (2014) [48] (if the sample size assumes a minimum compliance rate of 70% for the pilot RCT, a 50% sample size for the main trial is recommended, and Teere et al. (2014) also recommended a size of 60/group for a pilot study). Based on these recommendations for the pilot study, we defined a final sample size of 140 participants per arm, 70 in each group, and a total of 35 in each cluster. This sample size is large enough to meet the expectations of a pilot C-RCT and to account for potential loss to follow up.

## Enrolment

Voluntary informed consent will be collected from each study participant after screening for eligibility and randomization of the intervention and control areas. Consent will be obtained with a witness present in either written (language of choice) or oral form (if the participant is not literate). Individuals who are 13–17 years of age will provide consent with the addition of an assent form and a guardian based on context procedures for those within this age group. All participant medical records and personal information will be kept confidential to ensure privacy during the study. Only certified healthcare providers participating in the trial and project leads will have access to confidential records. The other project team members will have access to vital medical information pertaining to the trial, but another private participant information will be inaccessible.

### Allocation of health areas

Women attending the antenatal clinic and those identified by a community health worker will be screened for eligibility using a poverty assessment scale that will be employed within the study and incorporated into the Magpi data collection software (https://home.magpi.com/). Standard poverty assessment tools [49] will guide the development and adaption of the final tool to be employed. However, the study will consider contextual approaches of identifying those considered “poor” within the respective communities. Community health workers will help in identifying eligible pregnant women but will be blinded to the cutoff poverty scale that will be employed within the study for inclusion. The project team will decide on the cutoff for poverty eligibility, which will be primarily determined and informed by the formative study, taking context into consideration. Those individuals who are eligible and provide informed consent will be allocated to their respective groups by the project lead and/or coordinator. Upon entering the trial, participants will be aware of their intervention because the randomization is performed at the level of the health area, and it will not be possible to blind the participants.

### Steps in recruitment

Once a woman is identified by a community health worker to be pregnant, she will be asked a series of pre-eligibility questions after providing informed consent using the recruitment text. The woman will be allowed to ask questions to ensure that she has fully understood the project. Once the woman demonstrates an understanding of the informed consent form that will be administered by either the project lead or project coordinator, she will be asked to sign the form. A set of questions will be administered to screen for eligibility in terms of poverty assessment. These questions will be uploaded into Magpi, which will enable the project lead and coordinator to view the responses. For women who are identified by healthcare workers, a community health worker and project coordinator will follow up at home to administer the eligibility questions and to record the GPS location. Once a woman is identified by the project lead and coordinator to be eligible, the participant is reported to the community health worker or provider. Women will be recruited on a weekly basis until the sample size is attained. Once the participants are enrolled, they will be given a unique identifier number by the healthcare provider; in addition, a phone and a solar charger will be assigned to the woman in the intervention areas.

### Recruitment and follow up

Recruitment into the study will be prospective, and each participant will be followed during their pregnancy until they deliver. Mothers who do not deliver in the hospital will be followed-up in the community by community health workers within 1 week of delivery. The study will run for 18 months to allow time for the outcome to be measured and for enough participants to be enrolled in the study (this includes the formative phase). Recruitment is primarily based on referrals through community health workers, by word of mouth in the community, and through health-educational programs and healthcare workers in respective health clinics. Prior to introducing the intervention, community sensitization and the involvement of village leaders will be carried out in the health areas. Community meetings will be organized to explain the intervention to all community members through a brief pop-up presentation by the project lead and project coordinator. Furthermore, community health workers will be trained to inform all pregnant women about the intervention on an individual basis.

A total of eight community health workers will be involved in the project, two from each health area. Recruitment will occur within 8 months (allowing enough time for all women to deliver within the 18-month study time frame), and each community health worker will be responsible for recruiting approximately 35 pregnant women (approximately five women per month) to attain the estimated sample size. However, these districts have yearly deliveries totaling a minimum of 500 in Bali and over 1000 in the Ndop district [8]. Therefore, the estimated sample sizes are anticipated to be reached within a year. However, considering that all health areas within the district are eligible, the anticipated contingency plan for recruitment will be to recruit individuals from other health areas within the same district or divide the participating district into zones.

### Intervention and control

i. Intervention

The intervention is a prenatal management system (PNMS), which utilizes pictographs and local dialects by (i) using pictographs that reflect local norms and realities to communicate health needs and concerns, (ii) providing ongoing mobile family planning education in local dialects and sending reminder text messages, (iii) communicating emergencies to providers using geographic information system (GIS) and geo-localization, (iv) increasing access to and utilization of antenatal care and skilled delivery, and (v) integrating this technology into selected district health systems.

Upon registration into the PNMS, pregnant women will be provided five e-vouchers; one e-voucher will be automatically redeemed at each prenatal visit, and the account of the health center will be credited. Mobile phones will be provided to eligible pregnant women, along with electronic vouchers that will be redeemed upon antenatal visits and delivery. Each eligible pregnant woman will be assigned five vouchers (four for antenatal visits and one for delivery, including five transportation vouchers). The vouchers will be administered electronically, and the users of the application will communicate their state of health with providers using graphic signs on their phones or direct calls, which on arrival will be sent to the appropriate health personnel. Messages will consist of both audio and text messages. The health personnel will receive this information on their computers as well as on their phones as SMS alerts and will react appropriately.

Training of healthcare providers and community health workers in using the PNMS and training of pregnant women on how to use the features of the mobile application, including the GIS feature during emergencies, will be provided. The proposed interventions (e-vouchers and mobile phone application) are noninvasive and impose no direct adverse effects on the participants. Every woman is assigned a code to her profile that identifies her upon each visit. There are six graphical icons embedded within the app that indicate the following categories: pain, emergency, family planning, antenatal care, medical advice, and postnatal care.

#### Antenatal care package

The focused antenatal care package by the WHO (https://www.who.int/pmnch/media/publications/aonsectionIII_2.pdf) includes essential interventions in antenatal care as listed by the WHO, which include the identification and management of obstetric complications such as preeclampsia, tetanus toxoid immunization, intermittent preventive treatment for malaria during pregnancy (IPTp), and the identification and management of infections including HIV, syphilis, and other sexually transmitted infections (STIs). Pregnancy-related complications, particularly preeclampsia, will be diagnosed and managed. Identification and surveillance of the pregnant woman and her expected child will be conducted. Women and their families will be provided with appropriate information and advice for a healthy pregnancy, safe childbirth, and postnatal recovery, including care of the newborn, promotion of early, exclusive breastfeeding, and assistance with deciding on future pregnancies to improve pregnancy outcomes.

**Fig. 3:**
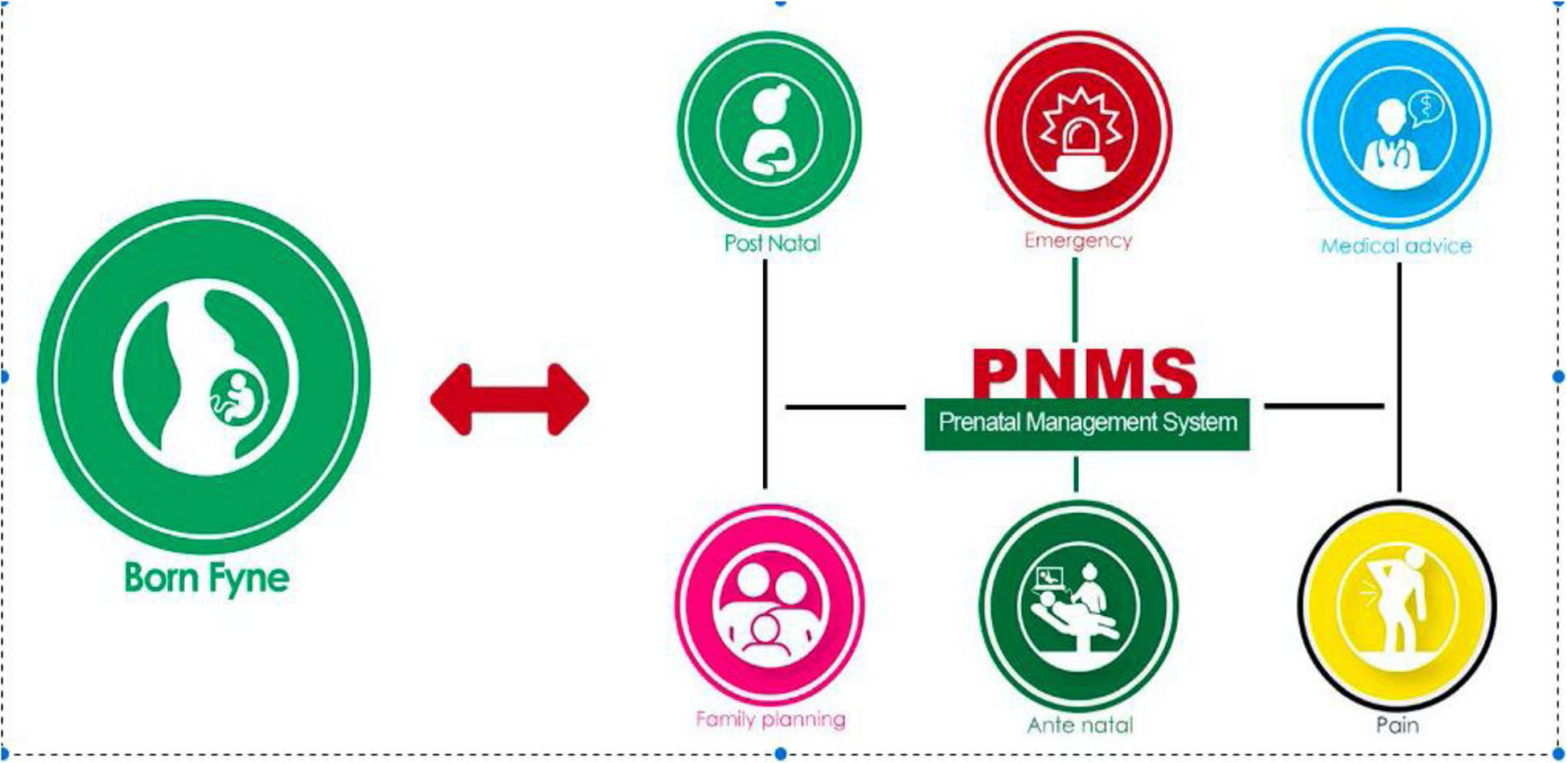
Graphical presentation of the pre-natal management system

The mobile application consists of both a user interface and web-based platform for providers. The user interface consists of the six icons as described below (see Fig. 3), which pop up on the participants’ mobile phone once the application is downloaded. Upon activating any of the icons at the point of need, the provider will be alerted via the online platform, which is the PNMS. The health care provider can collect patient information (participants) history and demographics, which will help the providers to follow up their patient (participant) based on their history for continuity of care. During antenatal care visits, as participants attend their antenatal care visit, the system will prompt the provider to document the visit and timing and to register the schedule for the next visit, which will auto-generate a reminder message that will be sent to the participant a few days before the next scheduled visit. This is the same protocol that will be used for any additional appointments and during delivery. The features of the mobile application and their specific functions are briefly described below.

#### Mobile family planning feature

This feature will be audio-recorded in the local language that is understood by everyone in the community, for example, “pidgin English.” In addition, mobile family planning messages will be recorded in their local dialect, and there will be options for listening to the message in multiple dialects. The final mobile family planning message will be developed based on data generated from the formative study.

#### Geographic information system feature

This is the emergency feature. It is also presented graphically on the participants’ mobile phones and activated during emergencies by simply touching the appropriate emergency icon on the screen. The emergency icon signals the provider with the location of the pregnant woman, and the provider or the service driver or an assigned individual can use geo-localization to navigate and reach out to the woman in distress. These individuals will be trained on how to use geo navigation to locate a distressed patient.

The other icons, namely pain and postnatal and medical advice, connect with the providers in the same way; specifically, participants touch the icon, which sends a signal to the provider. The participants can then discuss issues or ask questions as needed. In addition, the pain icon functions in a similar manner to the emergency icon.

ii. Active control group

This group is “business as usual” (standard practice). Women will pay out of pocket for their transportation to antenatal clinics, antenatal care visit services, and delivery. In addition, no mobile phones will be distributed to this group, and, therefore, they will not have access to the mobile family planning and geographic information system (GIS) features. Moreover, those participants who may own mobile phones will be unable to download the application during the pilot study.

### Outcomes

#### Primary outcomes

We used the criteria from Thabane et al. (2010) [50] to conduct feasibility studies to describe our primary and secondary outcomes. The primary outcomes are categorized as feasibility and clinical outcomes, which assess the feasibility of the processes involved and the scientific assessment as follows:

Feasibility outcomes include an assessment of randomization, contamination, enrollment rate, follow up, compliance rate, refusal rate, and adherence rate.

Clinical outcomes (planned for the larger study) include the number of antenatal visits, skilled birth delivery, family planning, and maternal mortality and failure rates.

#### Secondary outcomes

The secondary outcomes include operational and economic outcomes, which assess the resource utilization involved, and management of the software as follows: human resource capacity, equipment availability, district and health area capacities, project timeline, and cost. These outcomes are operationalized in Table 1.

**Table 1:** Table indicating feasibility, clinical, operational, and economic outcome variables

Outcome measures	Scale	Type	Measure	Method of analysis
Feasibility outcomes				
Evaluate randomization	Ordinal	Binary	% of health areas with successful stratification and randomization	Feasibility threshold of 95%
Enrolment rate	Nominal	Binary	% of participants with successful enrolment within 6 months into the trial	Feasibility threshold of 50%
Complete follow-up	Ratio	Continuous	% of participants who complete follow-up at 8months into the trial	Feasibility threshold of 80%
Compliance rate	Ratio	Continuous	% of participants who do not follow procedure as allocated	Feasibility threshold of 10%
Contamination	Ratio	Continuous	% of contamination	Feasibility threshold of 10%
Refusal rate	Ratio	Continuous	% of participants who refused to respond	Feasibility threshold of 5%
Adherence rate	Ratio	Continuous	% of participants who adheres to the intervention	Feasibility threshold of 80%
Clinical outcomes				
Failure rate	Ratio	Continuous	% of participants whom intervention (mobile phone features) failed to function according to design	*n* (%)
Mortality	Ratio	Binary	Number of maternal deaths	*n* (%)
Number of ANC visits	Nominal	Binary	Number of ANC visits attended	Mean (SD)
Number of skilled birth delivery	Nominal	Binary	Number of skilled birth delivery	Mean (SD)
Family planning	Nominal	Binary	% using family planning/awareness	*n* (%)
Operational outcomes				
Resource capacity	Nominal	Binary	Average number of human resources	Mean (SD)
Equipment availability	Nominal	Binary	Average number of equipment	*n* (%)
District and health areas capacity	Nominal	Binary	% of resource functionality and communication network	*n* (%)
Project timelines	Nominal	Binary	Average length of follow-up	Mean (SD)
Data collection time	Nominal	continuous	Average time to collect data	Mean (SD)
Matching data from other sources	Nominal	binary	% of success in matching data sources	*n* (%)
Data completeness	Nominal	Binary	% of participants who do not provide complete response	*n* (%)
Acceptability of using mhealth and e-vouchers by participants	Nominal	Binary	% of participants who accept the use mhealth	*N* (%)
Acceptability of using mhealth and e-vouchers by providers	Nominal	Binary	% of providers who accept the use of mhealth	*N* (%)
Use of transportation vouchers	Nominal	Binary	% of women using transportation vouchers	*N* (%)
Economic outcomes				
Total cost of care	Ratio	Continuous	Average total cost of care	Mean (SD)
Cost per woman enrolled	Ratio	Continuous	Average total cost of care per woman enrolled	Mean (SD)
Additional cost due to emergencies	Ratio	Continuous	% with additional emergency cost	*n* (%)

### Data collection

#### Quantitative approach

The data collection method will include regular follow up with community health workers and questionnaires. The number of women using transportation vouchers and the cost will be verified and recorded. All vouchers will be distributed and reimbursed electronically. All costs will be retrieved, and at the end of the study, the health economist in the study will assess the cost. A review of each clinic visits and assessment of compliance (e.g., antenatal care attendance, communications with communications, communications with physicians using mobile phones for follow up, and reminder text messages) will be assembled by the main project lead and research coordinator during the study.

#### Outcome measures

All outcomes will be assessed by self-reports, objective assessments, and face-to-face interviews. The clinical outcomes in Table 1 will be collected objectively by providers directly on the PNMS platform as participants attend their antenatal care visit, and they will also be collected from self-reports by data collectors as follows. All participants in the intervention and control groups will be assessed at the following time points: baseline (T0) at recruitment into the trial, midpoint (T1) at 32 weeks into the trial and endpoint (T2), which is immediately after delivery. For the postnatal period, 2 months immediately after delivery (post-intervention period), data will be collected solely on exclusive breastfeeding, which is at T3 (2 months after delivery). At each time point, participants will be asked the same set of questions plus repeated questions on antenatal care visit, skilled delivery and family planning. Baseline measures will include sociodemographic data on age, gender, income, education, marital status, place of residence, religion, and occupation.

The study timeline is outlined in Table 2, adapted from the Standard Protocol Items: Recommendations for Interventional Trials (SPIRIT) template [51], and data for the tertiary outcomes will be collected at the following time points: T0, T1, T2, T3, as defined above.

**Table 2:** Schedule of enrolment, interventions, and assessments in accordance with activities on WHO focused ANC package

TIMEPOINT^a^	June to August 2018	Study period (September 2018 to September 2019)							
	Formative study	Enrolment	Allocation		Post-allocation				Postnatal and Close-out
	Phase I		t0 (Baseline)	(ANC #1 (8–12 weeks)	ANC#2 (24–26 weeks)	t1 (ANC# 3 (32 weeks)	ANC#4	t2 Delivery	t3 Postnatal
Key informant interviews and focus group discussions	X								
Enrolment:									
Eligibility screen		X							
Informed consent		X							
Pre-assessment demographics		X							
Allocation			X						
Interventions									
[Group A e-vouchers and mobile phones]				X	X	X	x	X	X
[Group B control, standard care]				X	X	X	x	X	X
Assessments									
[medical history, examination, drug history, malaria/HIV screening, antenatal history, hep B screening, on ARV, preeclampsia, echography/fetal movements]			X	X	X	x	x	X	
[outcomes, #ANC, delivery, MMR,FP]			X		X		X		X
[Exclusive breastfeeding]									X
CHW weekly follow-up			X	X	X	x	x	X	X
Resource utilization and management	x	X	X	X	x	x	x	x	X
Economic analysis									X
Post Intervention FGD									X

Displayed under assessments

^a^List specific timepoints in this row

#### Baseline data for clinical outcomes

After enrolment, data collectors will collect data using a set of questions for both the intervention and control groups. The data collectors are blinded and will not know which area is the intervention area and which is the control area. Data collectors do not live in either the health area or the district and are not aware of the nature of the intervention.

#### Intervention

During the intervention period from October 2018 to September 2019, the following data will be collected: attendance at ANC1, ANC2, ANC3, and ANC4 visits and the timing of these antenatal visits, followed by a list of variables as outlined in Table 2.

Data will be collected at three different levels. At the hospital and/or health center level, data from the intervention group will be collected directly from the mobile web-based version online PNMS, where the doctor will log in to enroll the patients. The questionnaire will contain characteristics of the baseline data and an antenatal focused package. In the control areas, the data will be collected directly from the District Health Information System (DHIS2) as usual. Data collectors will collect data at different times using Magpi, and these data will be triangulated with the data that are collected by healthcare providers and through community health worker weekly follow-ups. The third level of data collection by community health workers will occur on a weekly basis, and this information will be collected using Magpi, which will allow geo-mapping of participants to help control for loss to follow up through displacement. These data will be triangulated for quality and consistency in both the intervention and control areas.

## Data management

Once data are collected in Magpi by data collectors and community health workers, the information will be immediately uploaded, and the project coordinator and research assistant will be able to view these data and check for any missing or erroneous data; any queries related to any data will be sent to the project lead to be resolved with the data collectors or community health workers who collected the data.

Attendance at antenatal care and delivery visits will be recorded in the web-based online prenatal system and will be checked online on a weekly basis by the project lead to control for any missing data, and any queries will be resolved by the district supervisor and the provider along with the project lead.

All attendance during the training and workshops, as well as stakeholder meetings, will be recorded in a handwritten format, and after each session, the participants will sign a feedback evaluation form designed specifically for participants attending the training sessions. Attendance during focus group discussions will be similarly recorded by hand, and records will be signed after each session. All key informant interviews and focus group discussion will be recorded with participant consent. Notes during the focus group discussion will be transcribed, and the recordings will be transcribed from pidgin English to standard English. All transcriptions will be checked for quality, and all photographs will be stored in the same package as the transcriptions. Consent forms and paper records will be stored in a locked cabinet in the office of the research coordinator, which has restricted access. All data will be stored in secured databases with restricted access.

One of the research assistants will be responsible for managing the quantitative data that will be analyzed by the statistician, and all data will be assigned a unique code to blind the statistician. Intervention and control areas will be assigned a unique code before making the data available to the statistician.

### Auditing

Regular auditing of the study conduct will be carried out by members of the team to verify whether the participants’ informed procedures have been followed correctly. Observation and assessment of community health worker weekly reports will be performed using a monitoring framework developed by the team, and participant attendance at antenatal care visits, as well as provider activities and supervision, will be monitored. Community health workers will attend group meetings with field coordinators monthly to review recruitment statuses, concerns, or issues brought forth by participants or potential study candidates, as well as other relevant study details. The community health workers will also attend all the workshops designed during the trial period. Problems discussed during these monthly meetings will be addressed appropriately. There are no safety concerns associated with the intervention.

### Data analysis

#### Quantitative approach

The data will be analyzed following the guidelines for Consolidated Standards of Reporting Trials (CONSORT) statements for cluster randomized trials [52]. The trial statistician will remain blinded until the main analyses are complete (i.e., he/she will not be involved in patient recruitment, data collection, and data management). Baseline characteristics will be presented separately for each randomized group. Baseline characteristics will also be presented separately for dropouts and those who complete the study within each treatment group. Descriptive statistics will be used to summarize patient demographics and to assess the baseline clinical characteristics of all study participants.

All analyses will be conducted at the end of the trial because the outcomes may not be readily measured, long follow-ups and multiple interventions are needed for maximum trial efficacy, and there are no severe side effects or other safety issues of concern that may warrant an interim data analysis. However, a sequential data analysis may be conducted during the midterm evaluation for reporting.

The study populations will be analyzed based on the intention-to-treat (ITT) approach to eliminate potential biases associated with nonrandom loss of participants (refusing to continue participating in the trial). Follow-up data will be collected for all participants, regardless of whether they drop out. Compliance for this study is expected to be good; however, missing data may influence the ITT analysis. Multiple imputations and mixed model repeated measures will be used to handle data missing at random.

#### Integrating qualitative and quantitative data

Data analysis for qualitative and quantitative data will be performed separately; however, inferences from both datasets will be used to draw conclusions. Data integration will be conducted using the embedded design approach as described by Palinkas et al. (2011). The qualitative data will help inform some components of the intervention from the outset in refining some features of the mobile application, and during and after the intervention, the post-qualitative data collection will help augment the outcomes of the intervention.

### Addressing sources of bias

#### Qualitative

The interviews will be audio-recorded to ensure accuracy of the data. Data will be coded by multiple team members for consistency. Constant comparison of codes will be performed by two members, and inter-rater reliability will be determined. Data will be triangulated to ensure validity.

#### Quantitative

After enrolment, community health workers will follow up with women on a weekly basis. We anticipate that performance bias will be reduced considering that the outcome is objective, and we also anticipate that detection bias will be reduced by blinding the statistician and data collectors. Providers, community health workers, and patients (pregnant women) will not be blinded because this is not possible due to the nature of the intervention. Selection bias will be eliminated through random assignment. Attrition bias may be likely in the control group because women in the control group may want to drop out of the study or refrain from providing or responding to questions during subsequent visits, especially because they will not be provided any mobile phones. To minimize this bias, we will sensitize the community through stakeholder meetings on the importance of the control group to encourage participation. To control for contamination, e-vouchers will be used, as the women will not be able to sell, misplace, or share their vouchers. Additionally, the use of equity scores as a baseline cutoff for rural or remote rural areas will help to control for contamination, as the health areas are far from each other. Prior to enrolment, the community sensitization, stakeholder meetings, and education programs will be performed to help increase compliance in the control group and prevent women from becoming unresponsive during the trial. We anticipate minimal loss to follow up due to the nature of the exposure (pregnancy) and outcome (antenatal care and skilled birth delivery).

### Economic data

We will conduct cost minimization evaluations for the intervention to identify feasible and appropriate cost minimization strategies for the project. This information will be used to inform the cost for a transition-to-scale phase. Changes in the resources utilized over time in the district’s health areas relative to the control areas will be calculated and used in conjunction with the costs of setting up and delivering the intervention in the communities. The net cost of program delivery per family will be calculated and will represent the incremental cost of providing the program relative to standard practice.

## Discussion

The outcome of the feasibility project is to introduce an innovative platform for RMNCH delivery called the BornFyne-PNMS project. The paper describes the feasibility study protocol for a cluster randomized control trial to study the effectiveness of e-vouchers and a mobile phone application in selected communities in the northwestern region of Cameroon in improving the access to and utilization of reproductive maternal and newborn child health.

The data collected during this study will allow us to better assess feasibility of the intervention, processes, and resources necessary to scale up and inform a larger trial and how the intervention can boost quality of care. In addition, the results or strategies can also be used by the PBF community to enhance the delivery of care and quality of care indicators, especially as this community is mostly focused on RMNCH. This will then inform policy on specific demand-side strategies that are relevant, especially as this intervention intends to use the PBF platform as a potential platform for sustainability.

### Ethics

Ethical approval for this study has been obtained from the North West regional delegation of health in Cameroon and the University of Ottawa Social Science Ethics board. Administrative clearance has been received from all the district medical officers in the respective districts, the Division for Health Operations Research (DROS) in Cameroon, and the regional delegation for health for the Northwestern region. In addition, administrative approval was also obtained from the Ministry of Public Health at the national level in Cameroon. The trial has been registered with the WHO Pan African Trial Registration.

### Dissemination

The results will be widely disseminated through formal and informal mechanisms. During the post-FGD, meetings will be held by participants to inform them of the results and to obtain their feedback and recommendations. We will inform participants in the control arm regarding the possibility of scaling up if we transition-to-scale. Providers in the control group will be informed about how the intervention will be incorporated into their community and the considerations for the scale up. The study findings will be presented to stakeholders at the district and regional levels and to the ministry of health at the national level in collaboration with the delegation of research (DROS) in Cameroon. The results will be presented to the performance-based financing regional and national offices in Cameroon.

The results will also be presented at regional, national, and international conferences and meetings, as well as presented to the funding agency. Manuscripts will be prepared by the team for submission to peer-reviewed journals, and other strategies will be used to disseminate the results through development agencies, consortia, and innovative competitions.

### Anticipated use of results

We anticipate using the results to inform a larger trial related to effectiveness, as well as to estimate the cost involved to inform a larger phase of the study.

### Limitations

Due to political instability, data and project interruption, delays may be experienced during the project. With the adoption of PBF as a national strategy in Cameroon, the trial may use a pragmatic approach; the proposed non-PBF district may become enrolled into PBF at the time of the intervention.

### Strengths

The team assembled to conduct the feasibility study has experience in designing and implementing mhealth interventions. Second, some of the team members understand the health system in the context of the study. In addition, the study will be implemented within the platform of PBF which provides a pragmatic approach to better inform possible integration of vouchers within the PBF platform for RMNCH. Approval from the district medical officers of the respective pilot districts provides an additional strength for a pilot study.

## Supplementary information

**Additional file 1: Figure S1.** Project Theory of Change (Logic Model).

**Additional file 2: Table S1.** Components of the theory of change and some examples (list not exhaustive of the examples of activities).

## Data Availability

All the data sets that will be generated in the study will be available through publications and will also be submitted to the funding agency. In addition, all relevant data will be available and uploaded to the trial registry accordingly.

## Abbreviations

CHW: Community health workers
GIS: Geographic information system
PNMS: Pre-natal management system
PBF: Performance-based financing
RMNCH: Reproductive maternal and newborn child health
