# Supplementary figures and images for: A protocol for a pilot cluster randomized control trial of e-vouchers and mobile phone application to enhance access to maternal health services in Cameroon
Source: Pilot Feasibility Stud. 2020 Apr 14;6:45. doi: 10.1186/s40814-020-00589-y (PMC7155248; doi:10.1186/s40814-020-00589-y)

**Project Theory of Change (Logic Model)**  
**Supplemental Figure 1**

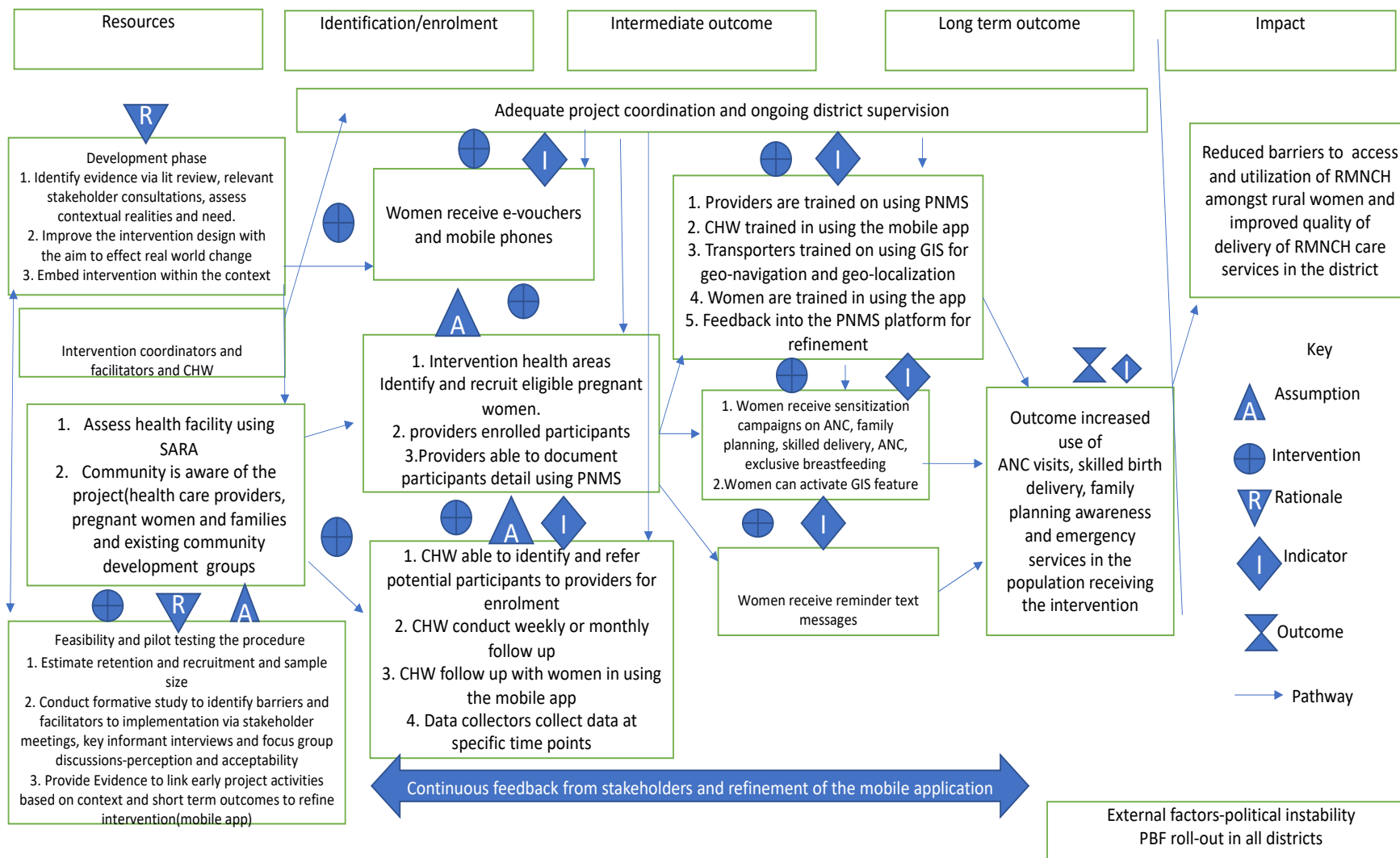

Supplement: Supplementary file 1 — Additional file 1: Figure S1. Project Theory of Change (Logic Model). [file 40814_2020_589_MOESM1_ESM.pdf]
