# Supplementary material for: A protocol for a pilot cluster randomized control trial of e-vouchers and mobile phone application to enhance access to maternal health services in Cameroon
Source: Pilot Feasibility Stud. 2020 Apr 14;6:45. doi: 10.1186/s40814-020-00589-y (PMC7155248; doi:10.1186/s40814-020-00589-y)
Supplement: Supplementary file 2 — Additional file 2: Table S1. Components of the theory of change and some examples (list not exhaustive of the examples of activities). [file 40814_2020_589_MOESM2_ESM.pdf]

Supplemental Table 1: Components of the theory of change and some examples (list not exhaustive of the examples of activities)

	Examples
**Assumptions**	Providers are engaged with the project and willing to document participants record using electronic platform (PNMS)<br>CHW are engaged in the program and willing to collect weekly and/or monthly follow up data as required for the project<br>Data collectors are engaged and willing to collect data at different time points
**Rationale**	Evidence from systematic reviews that mhealth can be effective in improving maternal health services<br>Evidence form the literature indicate cost is an impeding factor in accessing MNCH in Africa<br>Contextual evidence illustrates difficult in accessing and reaching health facilities during emergency
**Intervention**	Training of health care providers on using PNMS<br>Training of CHW on using the mobile app<br>Training of women on using the mobile app to communicate with providers<br>Women are provided e-vouchers and mobile phones<br>Awareness campaigns on family planning, ANC, skilled birth delivery, exclusive breastfeeding conducted by intervention coordinators<br>Women received reminder text messages,<br>Transporters trained on using GIS for geo navigation and geo-localization<br>Women received and can listen to uploaded family planning messages embedded within the app
**Indicators**	Number of providers trained on using PNMS<br>Number of CHW trained<br>Number of ANC visits<br>Number of skilled birth delivery<br>Number of participants enrolled<br>Number provided e-vouchers and mobile phones etc.
**Intermediate outcomes**	Increase awareness on family planning,<br>Change in providers attitude in using electronic platforms and mhealth
**Long term outcomes**	Improve access and utilization to emergency services in the intervention areas<br>Improve quality of care delivery in the intervention area<br>Reduce maternal mortality deaths in the intervention area
**Impact**	Improve access and utilization to emergency services in the district<br>Improve quality of care delivery in the district<br>Reduce maternal mortality deaths in the district
